# Intelligent Optimization of the Film-to-Fiber Ratio of a Degradable Braided Bicomponent Ureteral Stent

**DOI:** 10.3390/ma8115397

**Published:** 2015-11-11

**Authors:** Xiaoyan Liu, Feng Li, Yongsheng Ding, Ting Zou, Lu Wang, Kuangrong Hao

**Affiliations:** 1 Engineering Research Center of Digitized Textile & Apparel Technology, Ministry of Education, College of Information Science and Technology, Donghua University, Shanghai 201620, China; liuxy@dhu.edu.cn (X.L.); lifeng_ss@hotmail.com (F.L.); krhao@dhu.edu.cn (K.H.); 2 Key Laboratory of Textile Science and Technology, Ministry of Education, College of Textiles, Donghua University, Shanghai 201620, China; zoutingdhu@foxmail.com (T.Z.); wanglu@dhu.edu.cn (L.W.)

**Keywords:** bicomponent, ureteral stent, biodegradable, HSVRM model, PSO algorithm

## Abstract

A hierarchical support vector regression (SVR) model (HSVRM) was employed to correlate the compositions and mechanical properties of bicomponent stents composed of poly(lactic-*co*-glycolic acid) (PGLA) film and poly(glycolic acid) (PGA) fibers for urethral repair for the first time. PGLA film and PGA fibers could provide ureteral stents with good compressive and tensile properties, respectively. In bicomponent stents, high film content led to high stiffness, while high fiber content resulted in poor compressional properties. To simplify the procedures to optimize the ratio of PGLA film and PGA fiber in the stents, a hierarchical support vector regression model (HSVRM) and particle swarm optimization (PSO) algorithm were used to construct relationships between the film-to-fiber weight ratio and the measured compressional/tensile properties of the stents. The experimental data and simulated data fit well, proving that the HSVRM could closely reflect the relationship between the component ratio and performance properties of the ureteral stents.

## 1. Introduction

Ureters are a pair of narrow thick-walled tubes that carry urine from kidneys to urinary bladders. Trauma, congenital malformation, tumor and calculi could obstruct ureters. Ureteral stents area type of tubular medical device that repairs the obstructed or impaired ureters. However, most ureteral stents currently on the market are non-biodegradable, with disadvantages such as causing ureteral infection, sedimentation of calculi, waist pain, abdominal discomfort, blood urine, stent breakage, urine return and other symptoms [1,2]. A secondary surgery, which has the potential to cause complications, is usually required to remove the stents.

Much attention has been paid to developing biodegradable ureteral stents, which can be degraded and subsequently discharged after recovery of the impaired ureter and, thus, help avoid a secondary surgery [3,4]. However, the materials for assimilable ureteral stents should be biocompatible, controllably biodegradable with non-toxic degradation products and mechanically robust.

Biodegradable synthetic polymers are widely used to prepare biomedical devices due to their consistent quality [5], in addition to meeting all of the above-mentioned requirements. Poly(glycolic acid)(PGA),poly(lactic acid) (PLA) and poly(lactic-co-glycolic acid) (PGLA) have been widely used to prepare single-component ureteral stents. PGA and PLA are two types of biodegradable synthetic polymers that have been approved by the United States Food and Drug Administration (FDA) for clinical use. As an aliphatic polyester with a simple molecular structure, PGA has good biocompatibility, biodegradability, high crystallinity and good mechanical properties [6,7]. However, PGA has poor processability due to its high melting temperature. PGLA, the clinically-used copolymer, is synthesized by designated ratios of lactic acid and glycolic acid and, thus, has good biocompatibility and biodegradability, as well as controllable degradation profiles. Bergman *et al.* [8] repaired defective ureter using PLA films in animal models. Assimos *et al.* [9] reconnected canine ureters using PGA stents. Hou *et al.* [10,11,12] evaluated the biocompatibility and biodegradability of PGLA (20 LA:80 GA and 50 LA:50 GA) stents in animals and indicated that the drainage effects of the stents were satisfactory, and the degradation products could be discharged from urine without adversely affecting the excretional and secretional behaviors and the urination kinetics. However, relatively large residues of PGLA showed risks of obstructing ureter and adversely affecting kidney functions. In summary, using single-component stents for ureteral repair demonstrated many drawbacks, such as uncontrollable degradability, unsatisfactory mechanical properties and degradation.

In order to alleviate these problems mentioned above, two or more types of biodegradable synthetic polymers could be blended for the development of ureteral stents. Chew *et al.* [13,14] demonstrated satisfactory clinical effects in animal models using patented Utiprene stents produced with PLGA (80LA:20GA) and PEG (Pol Med). However, complicated preparation and inconsistency in the quality of stents restricted their wide applications. Wang *et al.* [15,16] fabricated a biodegradable ureteral stent using PGA and PGLA. Zou *et al.* [17] investigated the mechanical properties of five types of PGLA/PGA stents and indicated that the stents had both good compressional and tensile properties when the ratio of PGA over PLA was 1:1. 

It is difficult to complete the optimization experiments in the laboratory, as the processes are time consuming, expensive and labor intensive. Computational simulation could compensate by saving cost, time and labor. Using simulation, the mechanical properties of stents with different structures prepared under different conditions could be predicted, while the component ratio of PLA and PGLA and braiding parameters for the preparation of stents could be predicted as long as the required mechanical properties are proposed. A few examples have proven the effectiveness of simulation in the study of the mechanical properties of biomedical devices. Xiao *et al.* [18] presents the Support Vector Machine (SVM)-improved particle swarm optimization hybrid algorithm to bi-directionally forecast the productive process for carbon fiber, which can be considered as predicting the performance of carbon fibers and designing a method for the new carbon fiber production. A bi-directional prediction approach is developed to predict the production parameters and performance of differential fibers based on neural networks and a multi-objective evolutionary algorithm [19].

In this paper, a hierarchical support vector regression (SVR) model (HSVRM) was proposed to simulate the braiding process of PGLA/PGA bicomponent ureteral stents. The primary and high-level SVR models were used to simulate the process of mixing and braiding and the process of thermal treatment, respectively. In order to obtain the optimal component proportion for the bicomponent ureteral stent, performance evaluation indices were defined; meanwhile, the particle swarm optimization (PSO) algorithm was used to get the optimal mixing ratio.

This paper is organized as follows: Section 2 describes the synthesis of the principles of a multi-component stent and the HSVRM. Section 3 proposes a continuing reference deviation performance evaluation index, the ratio of the components to build the optimal search problem with the PSO algorithm to solve. Section 4 presents the results and the discussion, while Section 5 shows conclusions and future prospects.

## 2. Bicomponent Braided Ureteral Stent Modeling

### 2.1. Materials and Samples

**Figure 1:**
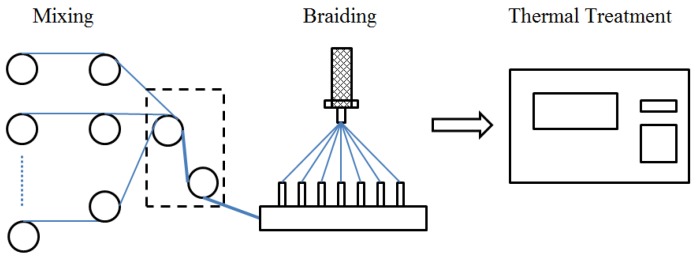
Flow chart for the preparation of the bicomponent ureteral stent.

PGA and PGLA multi-filament yarns were supplied by Shanghai Tianqing Biomaterials Co. Ltd. (Shanghai, China). PGLA was a co-polymer of lactic acid/glycolic acid at a mole ratio of 1:9. The commercial 6Fr double pigtail Percuflexs^®^ Plus biostable polyurethane stent was selected as the control for the comparison of the mechanical properties. Three different stents (Table 1) were braided on a 32 bobbins braiding machine in the Biomedical Textile Materials Research Laboratory of Donghua University. All of the stents were braided into the same structure, tension and braid angle around a central core made of a polytetrafluoroethylene (PTFE) cords to ensure that all the stents had a uniform lumen with an internal diameter of 1.6 mm. Yarn mixing was conducted before braiding (Figure 1). Subsequently, No. 2 and No. 3 prototypes were heated for 0.3 min at 210 °C, which was between the melting temperature of PGA and (225 °C) and that of PGA (205 °C). After treatment, the PGLA fibers melt to continuous film, while the PGA fibers retained their fibrous structure. Figure 1 demonstrates the preparation process of bicomponent ureteral stents.

**Table 1:** Compositions of the stents.

Samples	PGA (%)	PGLA (%)
1	100	0
2	0	100
3	50	50

**Figure 2:**
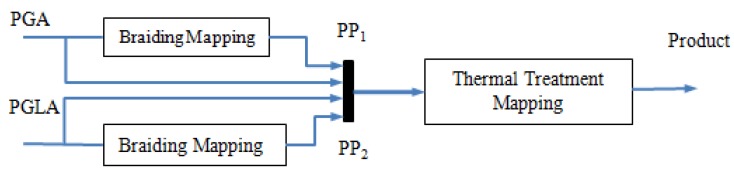
Mapping block diagram of the preparation process of the bicomponent ureteral stent.

The relationship between the properties of stents and each component was studied. The mechanical properties of the PLA/PGLA bicomponent stent are mainly attributed to that of the individual braided pure component stent (PCS) of PGA or PGLA. A particular mapping organization to quantify the relationship is demonstrated in Figure 2 derived from Figure 1. Figure 2 shows that in addition to the two PCS, fundamental characteristics of the raw materials were also embraced in the input stage of thermal treatment, due to the missing mechanical features of PGA and PGLA after braiding and thermal treatment.

### 2.2. Hierarchical SVM Model

**Figure 3:**
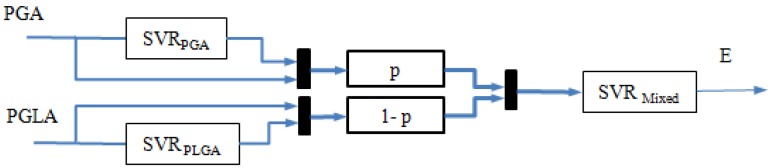
HSVRM block diagram.

As discussed above, mapping of the bicomponent stent preparation mainly consisted of two procedures, braiding mapping and thermal treatment mapping. The former can be modeled as a pure component model (PCM) and the latter as bicomponent model (BCM). By representing each mapping with a SVR model, a HSVRM could be obtained, as shown in Figure 3. The methods to establish these two models and the corresponding data preprocessing were also proposed.

In PCM, the modulus of elasticity PGA and PGLA should be obtained from experimental data of PGA and PGLA, so mechanical properties of these two PCSs, PGA and PGLA, can be simulated via regressing the property data obtained from lab testing. *n* is the dimension of the performance indexes. We used Δ*l* for radial deformation in the process of data sampling based on the experiments mentioned before and constructed a regression model for PCM using SVM [20].

The main idea is to build a nonlinear mapping $\text{Φ}:\;R^{n}\to \;H$, wherewe first map sampling points to a higher dimension feature space and then construct linear regression in this space to estimate the solution of the nonlinear regression problem in the original space. The form of regression estimate function is assumed as follows.

(1) y=f(x)=〈ω,Φ(x)〉+b

where ω is the weight vector and *b* is the bias. For precursor fiber PGA, *x* is ($e_{1},\;\text{Δ}l_{1}$) and *y* is $E_{p_{1}}$, and for precursor fiber PGLA, *x* is ($e_{2},\text{Δ}l_{2}$) and *y* is $E_{p_{2}}$. We introduce the penalty function and loss parameter *C* and ε. Solving the SVR model is equivalent to building the following quadratic programming problem.

(2) $$\min\;\Gamma (w)=\frac{1}{2}||\omega |{|}^{2}+C\sum_{i=1}^{l}\left(\xi_{i}+{\xi_{i}}^{*}\right)$$

(3) $$\begin{array}{ll} s.t.\; & \langle \omega ,\Phi (x_{i})\rangle \;+b-y_{i}\leq \xi_{i}+\varepsilon \\ & y_{i}-\langle \omega ,\Phi (x_{i})\rangle -b\leq {\xi_{i}}^{*}+\varepsilon \\ & \xi_{i},{\xi_{i}}^{*}\geq 0,\;i\geq 1,2,...,l \end{array}$$

In Equation (3), ${\text{ξ}}_{i}$ and ${\text{ξ}}_{i}^{*}$ are slack variables. We introduce a Lagrange function and dual variables. According to Karush-Kuhn-Tucke (KKT) conditions, this above problem can be converted into the dual problem as follows.

(4) $$\begin{array}{l} \min\;W({\alpha_{i}}^{\left(*\right)})=\frac{1}{2}\sum_{i,j=1}^{l}\omega_{ij}\langle \Phi (x_{i}),\Phi (x_{j})\rangle \\ \;+\varepsilon \sum_{i=1}^{l}\left({\alpha_{i}}^{*}+\alpha_{i}\right)-\sum_{i=1}^{l}y_{i}({\alpha_{i}}^{*}+\alpha_{i}) \end{array}$$

(5) $$\begin{array}{ll} s.t.\; & \sum_{i=1}^{l}\left(\alpha_{i}-{\alpha_{i}}^{*}\right)=0\\ & 0\leq {\alpha_{i}}^{\left(*\right)}\leq C,\;i=1,2,...,l \end{array}$$

where ${\text{ω}}_{ij}=\left({\text{α}}_{i}^{*}\;–\;{\text{α}}_{i}\right)\left({\text{α}}_{j}^{*}\;–\;{\text{α}}_{j}\right)$.

With the solution of Equations (4) and (5), we introduce the inner product kernel function *K* (*x_i_*, *x*). Then, Equation (1) can be rewritten in the following form.

(6) $$f(x)=\sum_{i=1}^{l}\left({\alpha_{i}}^{*}-\alpha_{i})K(x_{i},x\right)+b$$

Most of the values of (${\text{α}}_{i}^{*}\;–\;{\text{α}}_{i}$) in the last-written function are zero. The non-zero points constitute the so-called support vectors. The result of solving the model is the SVR model of ($e_{1},\text{Δ}l_{1}\;\to \;E_{p_{1}}$) and ($e_{2},\text{Δ}l_{2}\;\to \;E_{p_{2}}$).

For a bicomponent model, we can use the same means of SVR modeling, but it is worth noting that the independent variable *x* is defined as an augmented matrix.

(7) $$\tilde{x}=A\cdot [e_{1},E_{p1},e_{2},E_{p2},\Delta l]'$$

where *A* = *diag*({*p*, *p*, 1 – *p*, 1 – *p*, 0}), and *p* is the proportion of the first component.

### 2.3. Filter Design 

Due to the influence of mechanical wear and the observation environment, the data obtained from sampling had noise, which may increase the complexity and affect the accuracy of modeling. Therefore, data preprocessing is necessary to obtain a smooth curve.

We assume that an observation signal *x*(*n*) has the form as follows.

(8) x(n)=s(n)+v(n)

where *s* (*n*)is the effective signal, *v*(*n*) is the noise signal. In order to obtain the optima estimation *Ŝ* of effective signal *s*, we need to design a filter to minimize the mean square error between *S* and *Ŝ*.

(9) $$\min\{E{\left\|\hat{s}-s\right\|}^{2}\}$$

The process of *Ŝ* is derived from Equation (9) is known as the Wiener filter [21]. The impulse response of the linear filter is assumed as *h*(*n*). Then, the output of the linear filter is shown as follows.

(10) $$\hat{s}(n)=\sum_{m=-\infty }^{\infty }h(m)x(n-m)$$

The minimum mean square error criterion *h*(*n*) should meet the following regular equation.

(11) $$R_{xx}h=r_{xs}$$

where *R_xx_* is the *N* order autocorrelation matrix of signal *x*(*n*) and *r_xs_* is the cross-correlation function vector of *x*(*n*) and *S*(*n*). If *R_xx_* is a non-singular matrix, we can obtain the following function from Formula (11).

(12) $$h=R_{xx}^{-1}r_{xs}$$

The filter was applied to the obtained experiment data to achieve the smooth fitting data.

## 3. The Optimal Mixing Ratio

Ideal ureteral stents should have high tensile modulus and strength, in addition to being biocompatible for convenient implantation [22,23]. During the recovery of the impaired ureter, the stent will be squeezed by surrounding tissues and broken calculi and, thus, necessitates certain compressional properties. About 50% of ureteral stents failed since they could not afford the pressure around them [24]. In addition, the stents should have acceptable tensile properties to assure that they could be withdrawn without breakage, once inflammation occurred.

### 3.1. Property Evaluation of Bicomponent Ureteral Stents

A suitable standard is of critical importance to the fabrication of bicomponent ureteral stents. To the best of our knowledge, there has been no standard to evaluate the mechanical properties of ureteral stents till now. In general, if the designed stent has the same compressional and tensile properties compared to a commercial non-biodegradable stent, it is acceptable. Being overly stiff, stents might cause discomfort due to high friction with adjacent tissues. The following performance indices are defined to measure the features of the ureteral stent.

Definition 1. Sampling point performance indicators: Under the same test conditions, the similarity of the mechanical properties of testing stent *E_i_* and commercial stent *E_c,I_* is known as sampling performance. It is formed as below:

(13) $$J_{i}=K\cdot \exp[-\frac{1}{2}{\left(E_{i}-E_{c,i}-\mu \right)}^{T}\Sigma^{-1}(E_{i}-E_{c,i}-\mu )]$$

where *K* is a constant greater than zero. We introduce a tolerance margin μ into the above formula. In other words, the performance of stents is optimal if the formula *E* = *E_C_* + μ can be satisfied. Σ is the covariance matrix of different mechanical properties. Therefore, when *K*=1, the formula $J_{i}\in \left[0,K\right]$ is the normalized form of Equation (13).

Definition 2. The performance of stents being tested: the performance evaluation function *J* of stents being tested is defined as the root-mean-square of the performance of all sampling points of stents being tested.

(14) $$J=[\frac{1}{N}\sum_{i=1}^{N}{\left(J_{i}\right)}^{2}{]}^{1/2}$$

where *N* is the length of test data. Large *J* indicates that the mechanical properties of stents are close to the optimal performance of commercial stents. 

The performance evaluation function is strictly optimal. The optimized performance index can indicate consistency in the properties of the stents and the commercial stents throughout the testing process.

### 3.2. Problems in the Optimization of the Component Proportion

Currently, the optimization of component proportion is conducted using lab experiments, which is time consuming and not capable of predicting the performance properties of products. Therefore, constructing a reasonable model for optimizing the component proportion has a practical importance. According to Model (6) in Section 2 and Definition (14) in Section 3.1, given a set of model parameter ω and corresponding commercial stents performance *E_c_*, the problems in the optimization of component proportion can be expressed in the following form.

(15) $$p^{*}=\arg\underset{p}{\min}(J(p,E_{c}|\omega ))$$

(16) s.t.  0≤p≤1

Solving Equation (15) is a nonlinear optimization problem. Because of the complexity of the preparation process model and the performance evaluation function, it is unable to obtain the analytic solution of Equation (15). Therefore, it is not applicable to use traditional methods to calculate extreme points. Hereinafter, we will use the PSO algorithm to find out the optimal component proportion.

### 3.3. Searching the Optimal Solution via PSO

Particle swarm optimization was proposed by Kennedy and Eberhart [25]. The kernel of PSO is to simulate the social behavior of a flock of wild geese and to search the solution space according to guiding principles. In the algorithm of PSO, the status $X_{i}\in \;R^{B}$ of each particle *i* means a solution in *B* dimensional solution space. The flying speed of each particle is $V_{i}\in \;R^{B}$. A fitness function $f:\;R^{B}\to R$ is constructed. The smaller the value of *y* = *f*(*x*), the higher the fitness, the more superior the status of the particle is. We mark the historical optimal status of each particle as $p_{i,best}$ and the optimal status during the whole process among all particles as *g_best_*; the status update function of particle *i* is formed as follows.

(17) $$X_{i}^{k+1}=X_{i}^{k}+V_{i}^{k+1}$$

(18) $$V_{i}^{k+1}=\eta V_{i}^{k}+c_{1}(p_{i,best}-X_{i}^{k})+c_{2}(g_{best}-X_{i}^{k})$$

Here, η is inertia weight and $c_{1}$ and $c_{2}$ denote respectively the influence by self-experience and social experience. The second and third terms of the right side of Equation (18) denote respectively the flying direction of the particle tending to self-optimal status and global optimal status. The PSO algorithm has made a great contribution to continuous problems since it has been promoted.

The unknown proportion ratio *p* is treated as the status of a particle. Thus, Equation (15) can be converted to a function optimization problem with one dimension. According to the definition of the fitness function, in order to calculate in a user-friendly manner, we use the opposite value of the performance index as our fitness function, which is shown as follows.

(19) $$fitness(X_{i})=-J=-[\frac{1}{N}\sum_{i=1}^{N}{\left(J_{i}\right)}^{2}{]}^{1/2}$$

In order to guarantee that the particle searching space is satisfied with constraint Equation (16), we introduce a penalty term into the above fitness function and obtain the following form.

(20) $$fitness(X_{i})=-J+\Lambda$$

Here:

(21) $$\Lambda =\left\{ \begin{array}{ll} e^{\left\|x-0.5\right\|}\; & if\;\left\|x-0.5\right\|>0.5\\ 0 & otherwise \end{array}\right.$$

## 4. Experiments and Simulations

We did lab experiments and related simulation to verify the effectiveness of the proposed model. Firstly, data obtained from lab experiments were smoothed by the Wiener filter, as introduced in Section 2.3. Secondly, cross-validation was used to determine related parameters of the process model. Finally, using commercial stents as a reference, we obtained the optimal component proportion of bicomponent urethral stents by the HSVRM with the PSO algorithm.

### 4.1. Experiments and Data Smoothing

Tensile properties of stents were tested according to the ISO 7998-1998 standard for testing the tensile property of artificial blood vessels. We made three sets of tests respectively of the radial compressive property and the axial tensile property of stents being tested. The radial compression properties were measured using a radial compression apparatus, which was specially designed and built (Model LLY-06D, Laizhou, China). We compress the artificial blood vessel using a probe with a similar diameter and record the *in situ* displacement and force using transducers. The testing procedures for the radial compressional properties are as follows:

(1) Place a specimen with a length of 5 cm on the stand transversely;
(2) Compress the specimen at a speed of 10 mm/min until 50% of its diameter. The maximum force is considered as the radial compressional force;
(3) After a time period of 10 s, withdraw the probe at a speed of 10 mm/min. The percent of elastic recovery is defined as the ratio of the sum of immediate elastic deformation and delayed elastic deformation over the original diameter of the stent;
(4) Each data point is obtained from three tests.

The axial tensile properties were measured on a Darong universal mechanical tester (Model YG-B026H, Wenzhou, China). It gave experimental results for tensile strength (MPa) and elongation at break (%) [17]. All of the tests were conducted under standard environmental conditions (20 ± 1 °C RH 65% ± 2%). We used the average of the experimental data as a consequence. The experimental environment and parameters are shown in Table 2.

**Table 2:** Experimental environment and parameters.

Experimental Environment and Parameters	Radial Compressive Property	Axial Tensile Property
Length × inner diameter (mm × mm)	50 × 1.6	50 × 1.6
Compression/elongation (mm/min)	10	50
Temperature (°C)	25	25
Relative humidity (%)	65	65

**Figure 4:**
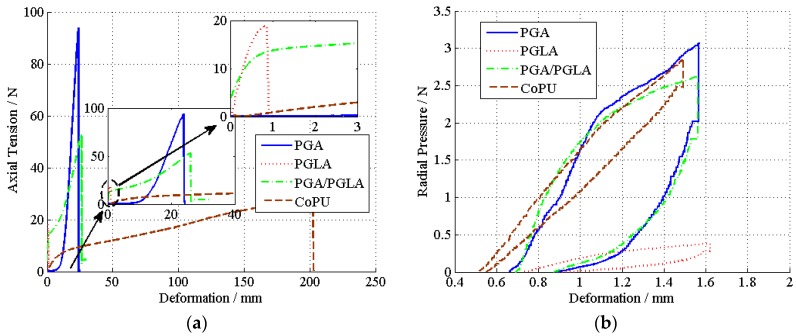
The strain forces corresponding to their deformations, axial tension is shown in (**a**) and radial compressure is shown in (**b**). The strain forces corresponding to their deformations.

From Figure 4, it can be seen that the deformation of the stent is a non-linear an elastic deformation. If the tensile strain were used as the *x* axis and the tensile stress as the *y* axis, a one-to-many unspecified reflection would be expected. However, the deformation of the sample is a function of time, and the sampling time is kept constant. Therefore, changes in two tensile and compressive properties can be converted to functions of time. 

**Figure 5:**
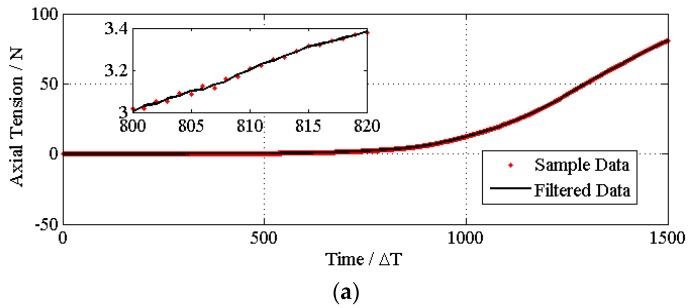

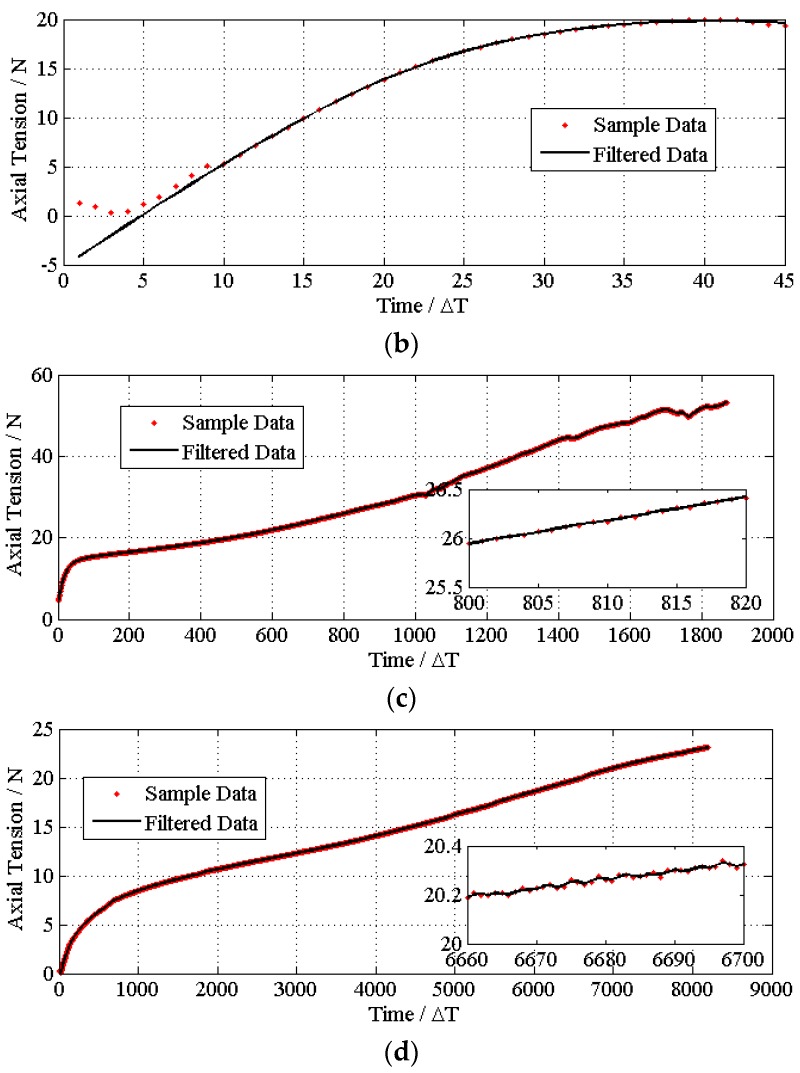
Data filter for each test. The samples data and filtered curves of the tensile strength are shown for PGA (**a**), PGLA (**b**), PGA/PGLA (50/50) (**c**) and commercial PU (**d**), respectively.

**Figure 6:**
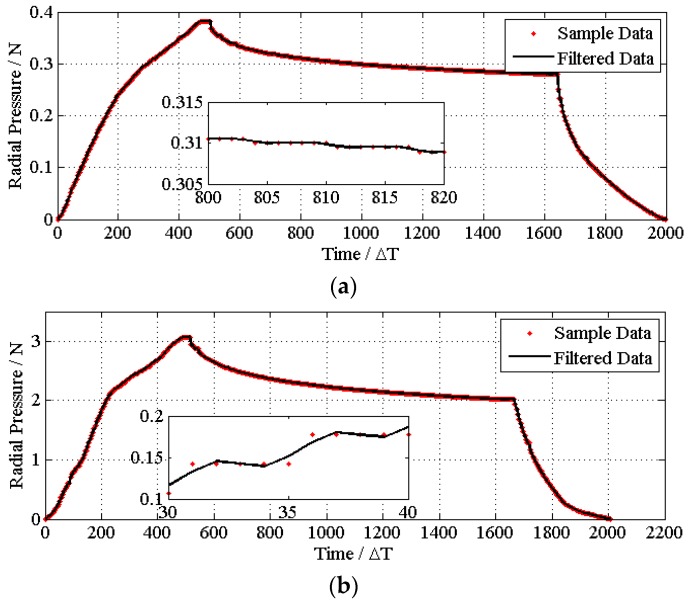

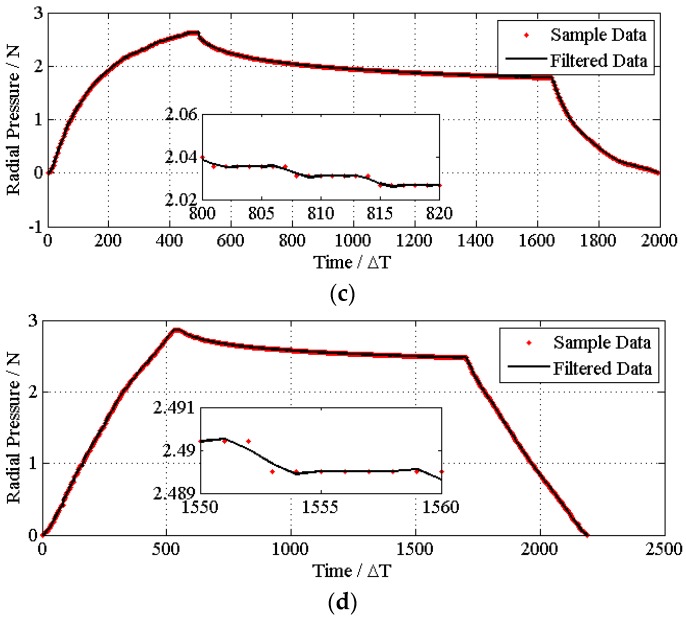
Data filter for each test. Data and filtered curves of the compressing performances are shown for PGA (**a**), PGLA (**b**), PGA/PGLA (50/50) (**c**) and commercial PU (**d**), respectively.

The time series of each stent regarding the axial tensile property and radial compression and their corresponding curve smoothed by the filter are shown in Figure 5 and Figure 6, respectively. The designed filter effectively removes the noise from the sampling system.

We magnify the area that contains violently-changed data points to demonstrate that after filtering, the curves become smoother. The nonlinear relation between strain and distortion can be easily observed from the experiment data. Therefore, if we use the linear fitting method, we would have lost many of the mechanical properties that needed to be considered.

### 4.2. Determining the Process Parameters

**Figure 7:**
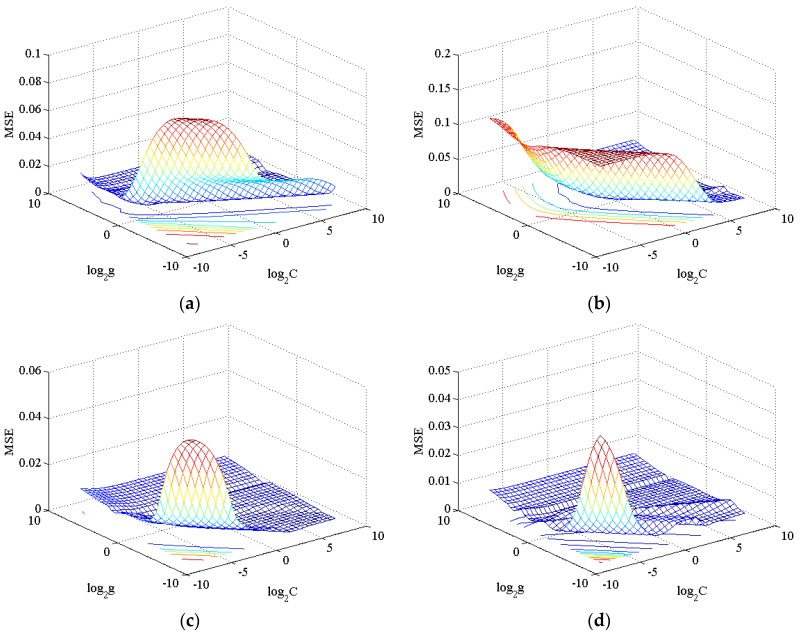
MSE surfaces of the cross-validation process for the SVR_PGA_ (**a**), SVR_PGLA_ (**b**), SVR_Mixed_ (**c**) and SVR_CoPU_ (**d**), respectively.

**Figure 8:**
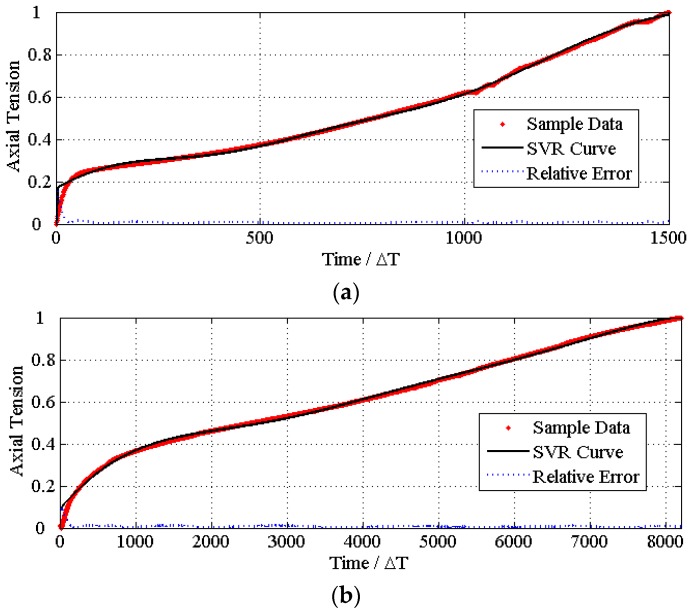

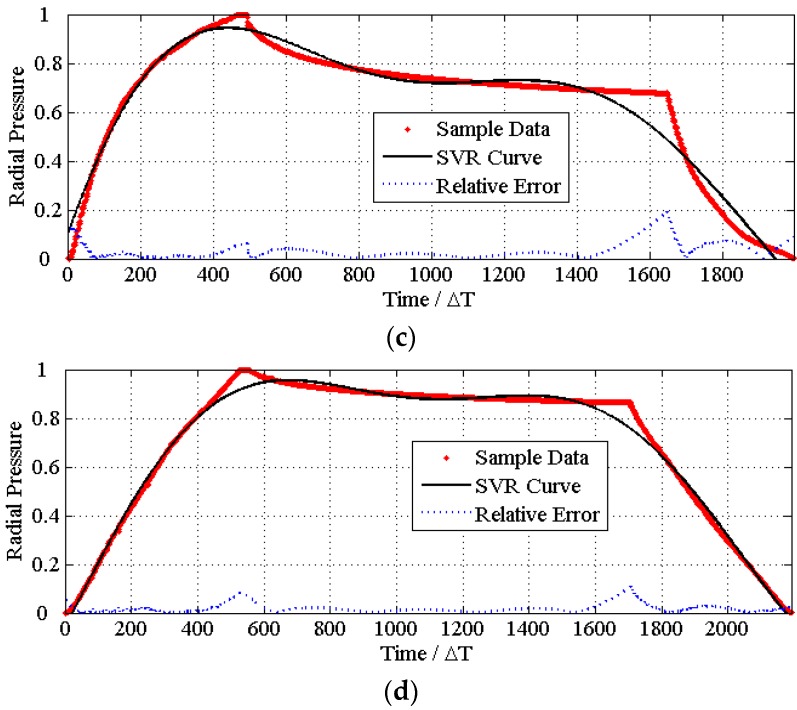
The time series of the ureteral stent’s properties. (**a**,**b**) The axial tension properties with the regression errors of SVR_Mixed_ and SVR_CoPU_, respectively; (**c**,**d**) the radial pressure properties with the regression errors of each.

According to the model in Section 2, the penalty coefficient *C* and the kernel function coefficient *g* need to be determined. Here, we select the radial base function (RBF) as the kernel function and the parameter g as the kernel bandwidth. The penalty coefficient controls empirical risk and reaches a compromise between empirical risk and confidence range. The bigger *C* is, the higher the fitting degree is and the smaller the error is. However, we should also consider that being oversized may lead to an over-fitting of training data. Kernel bandwidth *g* controls the sensitivity of the SVM to the change of input data. The smaller this value, the less sensible SVM is in the change of input data. The optimal parameters of the one-component model and hierarchical mixture model from cross-validation are shown in Table 3. The mean square error surfaces and fitting curves of normalized training data are shown in Figure 7 and Figure 8, respectively.

**Table 3:** Optimal parameters of SVR.

Parameter	SVR_PGA_	SVR_PGLA_	SVR_Mixed_	SVR_CoPU *_
*C*	1.4142	2.8284	0.0625	0.35355
*g*	4	0.70711	5.6569	0.25

* The SVR_CoPU_ only concerns the sampling time.

### 4.3. The Convergence of the PSO Algorithm.

Before using the PSO algorithm to obtain the optimal proportion ratio p^*^, we still need to determine PSO’s related parameters. The values of the related parameters are shown in Table 4.

**Table 4:** The parameter values of the PSO algorithm.

Population	Max Generation	η	$c_{1}$	$c_{2}$
200	100	0.6	1.2	1.2

According to the analysis in Section 2, the bicomponent proportion ratio *p* only influences the inputs of the SVR_Mixed_ section in the HSVRM and is independent of the first layer SVR. In order to reduce the computational burden, SVR_PGA_ and SVR_PGLA_ generate 100 sets of mechanical properties of PGA and PGLA in accordance with the time series. The original searching problem can be converted to such a situation, that when two raw material components and related pure component stents and a set of reference mechanical property are given, we need to solve proportion ratio p* to minimize the fitness function. Because the stretching test and compression test are conducted independently, the covariance of these two mechanical properties is zero. In order to reduce the computational burden, we may as well assume that the covariance matrix ∑ = *I*.

**Figure 9:**
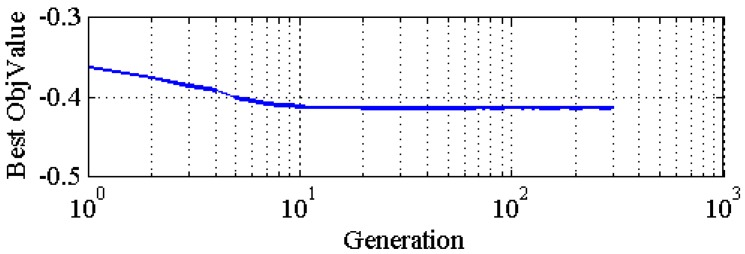
The evolution of global fitness.

By operating the above optimizing process 10 times independently, the average property when the algorithm has good convergence is calculated and set as p*. Figure 8 and Figure 9 are the evolution curves of the best individual’s fitness among their populations, respectively. The mechanical properties compared to the commercial PU are presented in Figure 9. The corresponding statistics data are listed in Table 5.

**Table 5:** The statistics data for the PSO algorithm.

Parameter	Mean	Standard Error
Convergence Iteration	288	6.187 × 10^‒6^
p*	0.6989	0.3517
fitness	−0.4133	0.0270

**Figure 10:**
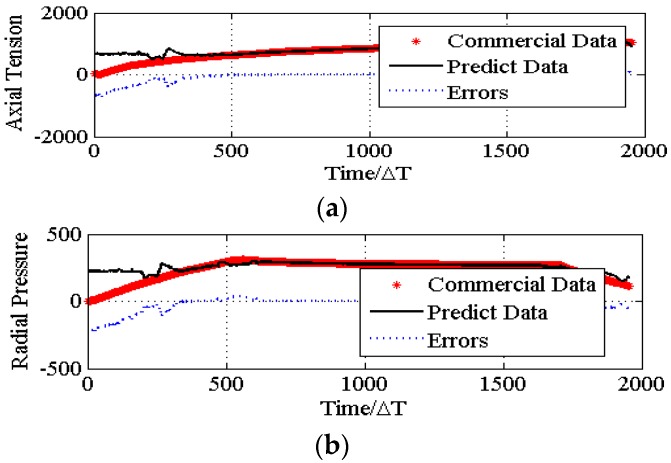
The mechanical properties’ comparison.

From Figure 10 and Table 5, it can be easily seen that using the optimal solution p* obtained from the PSO algorithm to produce urethral stents, the products can have uniform performances in the stretch interval (500, 2000) and the compression interval (425, 1850), respectively. The larger error in (100, 300) is due to the tremendous changes of testing data and the higher risk of fitting in this section. 

## 5. Conclusions

In this paper, we established an HSVRM to estimate the mechanical properties of PGLA/PGA bicomponent braided ureteral stents. In our simulations, the braiding process of each material was treated as a primary SVR, and the thermal treatment is replaced by a high level SVR with the input consisting of the materials and the PCSs mixed at the ratio *p*. In order to evaluate the performance of the ureteral stents, a novel performance index was first defined based on the deviation in the properties of the commercial stent. Unlike the empirical method, the proposed performance index cannot only guarantee the terminal feature of the products, but also force the transition performance to converge to the reference performance. Furthermore, the PSO algorithm was employed to search the optimal ratio of the two components for the stent preparation. 

In this work, we only considered static mechanical properties, which may not be adequate to characterize medical devices. In the future, dynamic performance properties will be investigated and simulated using improved models.
